# 5-Bromo-3-(4-fluoro­phenyl­sulfin­yl)-2-phenyl-1-benzofuran

**DOI:** 10.1107/S1600536812044054

**Published:** 2012-10-31

**Authors:** Hong Dae Choi, Pil Ja Seo, Uk Lee

**Affiliations:** a Department of Chemistry, Dongeui University, San 24 Kaya-dong, Busanjin-gu, Busan 614-714, Republic of Korea; b Department of Chemistry, Pukyong National University, 599-1 Daeyeon 3-dong, Nam-gu, Busan 608-737, Republic of Korea

## Abstract

In the title compound, C_20_H_12_BrFO_2_S, the dihedral angles between the mean plane [r.m.s. deviation = 0.006 (2) Å] of the benzofuran fragment and the pendant 4-fluoro­phenyl and phenyl rings are 84.98 (5) and 40.98 (6)°, respectively. In the crystal, mol­ecules are linked by C—H⋯O and C—H⋯π inter­actions.

## Related literature

For background information and the crystal structures of related compounds, see: Choi *et al.* (2009 ▶); Seo *et al.* (2011 ▶).
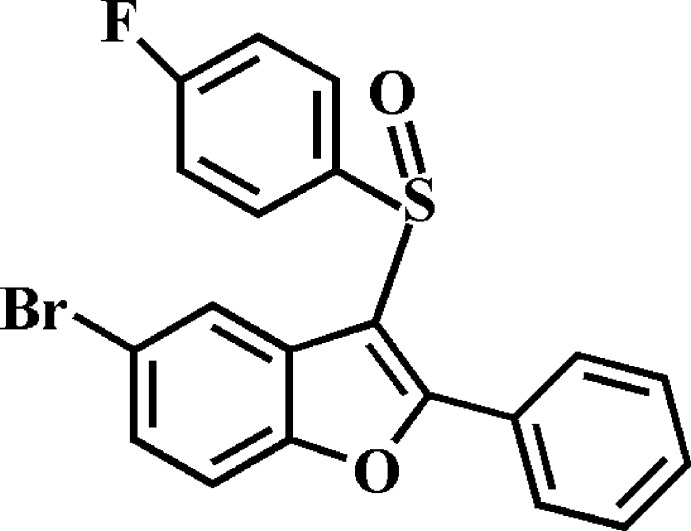


## Experimental

### 

#### Crystal data

C_20_H_12_BrFO_2_S
*M* *_r_* = 415.27
Triclinic, 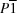

*a* = 9.2288 (2) Å
*b* = 9.4790 (2) Å
*c* = 10.4939 (2) Å
α = 67.396 (1)°
β = 89.933 (1)°
γ = 82.373 (1)°
*V* = 838.74 (3) Å^3^
*Z* = 2
Mo *K*α radiation
μ = 2.60 mm^−1^
*T* = 173 K
0.39 × 0.33 × 0.22 mm

#### Data collection

Bruker SMART APEXII CCD diffractometer
Absorption correction: multi-scan (*SADABS*; Bruker, 2009 ▶) *T*_min_ = 0.557, *T*_max_ = 0.746
15136 measured reflections
3888 independent reflections
3417 reflections with *I* > 2σ(*I*)
*R*_int_ = 0.045

#### Refinement

*R*[*F* ^2^ > 2σ(*F* ^2^)] = 0.030
*wR*(*F* ^2^) = 0.079
*S* = 1.05
3888 reflections
226 parameters
H-atom parameters constrained
Δρ_max_ = 0.65 e Å^−3^
Δρ_min_ = −0.74 e Å^−3^

### 

Data collection: *APEX2* (Bruker, 2009 ▶); cell refinement: *SAINT* (Bruker, 2009 ▶); data reduction: *SAINT*; program(s) used to solve structure: *SHELXS97* (Sheldrick, 2008 ▶); program(s) used to refine structure: *SHELXL97* (Sheldrick, 2008 ▶); molecular graphics: *ORTEP-3* (Farrugia, 1997 ▶) and *DIAMOND* (Brandenburg, 1998 ▶); software used to prepare material for publication: *SHELXL97*.

## Supplementary Material

Crystal structure: contains datablock(s) global, I. DOI: 10.1107/S1600536812044054/bv2213sup1.cif

Structure factors: contains datablock(s) I. DOI: 10.1107/S1600536812044054/bv2213Isup2.hkl

Supplementary material file. DOI: 10.1107/S1600536812044054/bv2213Isup3.cml

Additional supplementary materials:  crystallographic information; 3D view; checkCIF report

## Figures and Tables

**Table 1:** Hydrogen-bond geometry (Å, °) *Cg* is the centroid of the C9–C14 phenyl ring.

*D*—H⋯*A*	*D*—H	H⋯*A*	*D*⋯*A*	*D*—H⋯*A*
C10—H10⋯O2^i^	0.95	2.41	3.341 (3)	167
C19—H19⋯O1^ii^	0.95	2.50	3.447 (2)	175
C16—H16⋯*Cg* ^iii^	0.95	2.99	3.832 (2)	148

Symmetry codes: (i) 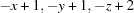; (ii) 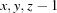; (iii) 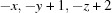.

**Fig. 1.**
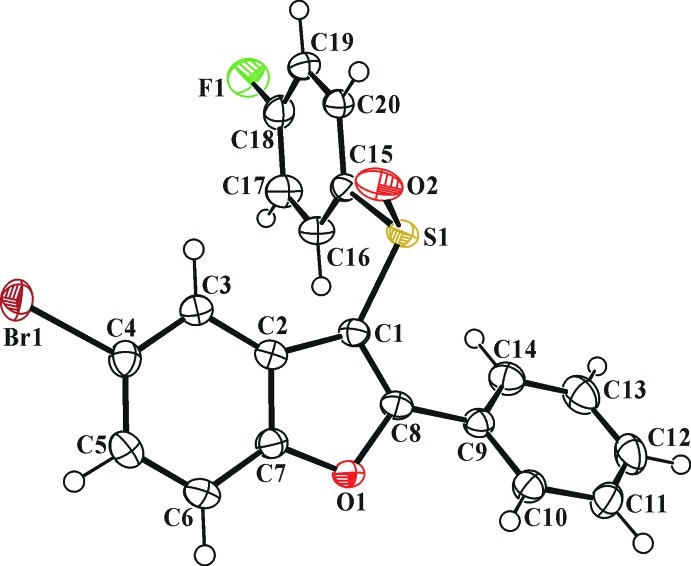
The molecular structure of the title compound with the atom numbering scheme. Displacement ellipsoids are drawn at the 50% probability level. H atoms are presented as small spheres of arbitrary radius.

**Fig. 2.**
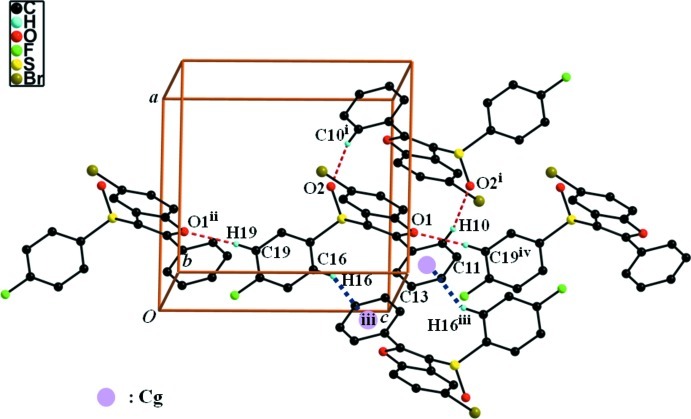
A view of the C–H···O and C–H···π interactions (dotted lines) in the crystal structure of the title compound. H atoms non-participating in hydrogen-bonding were omitted for clarity. [Symmetry codes: (i) - x + 1, - y + 1, - z + 2; (ii) x, y,z - 1; (iii) - x, - y + 1, - z + 2; (iv) x, y, z + 1.]

## supplementary crystallographic information

### Comment

As a part of our ongoing studies of 5-bromo-1-benzofuran derivatives containing 2-phenyl-3-phenylsulfinyl (Choi *et al.*, 2009) and 2-(4-fluorophenyl)-3-phenylsulfinyl (Seo *et al.*, 2011) substituents, we report herein the crystal structure of the title compound.

In the title molecule (Fig. 1). the benzofuran unit is essentially planar, with a mean deviation of 0.006 (2) Å from the least-squares plane defined by the nine constituent atoms. The dihedral angles formed by the mean plane of the benzofuran fragment and the pendant 4-fluorophenyl and phenyl rings are 84.98 (5) and 40.98 (6)°, respectively. In the crystal structure (Fig. 2), molecules are connected by weak C—H···O and C—H···π interactions (Table 1, Cg is the centroid of the C9–C14 2-phenyl ring).

### Experimental

3-Chloroperoxybenzoic acid (77%, 202 mg, 0.9 mmol) was added in small portions to a stirred solution of 5-bromo-3-(4-fluorophenylsulfanyl)-2-phenyl-1-benzofuran (319 mg, 0.8 mmol) in dichloromethane (30 mL) at 273 K. After being stirred at room temperature for 5h, the mixture was washed with saturated sodium bicarbonate solution and the organic layer was separated, dried over magnesium sulfate, filtered and concentrated at reduced pressure. The residue was purified by column chromatography (hexane-ethyl acetate, 2:1 v/v) to afford the title compound as a colorless solid [yield 63%, m.p. 445-446 K; *R*_f_ = 0.75 (hexane-ethyl acetate, 2:1 v/v)]. Single crystals suitable for X-ray diffraction were prepared by slow evaporation of a solution of the title compound in acetone at room temperature.

### Refinement

All H atoms were geometrically positioned and refined using a riding model, with C–H = 0.95 Å for the aryl H atoms. *U*_iso_(H) = 1.2*U*_eq_(C) for the aryl H atoms.

### Crystal data

**Table:**

C_20_H_12_BrFO_2_S	*Z* = 2
*M**_r_* = 415.27	*F*(000) = 416
Triclinic, *P*1	*D*_x_ = 1.644 Mg m^−^^3^
Hall symbol: -P 1	Melting point = 446–445 K
*a* = 9.2288 (2) Å	Mo *K*α radiation, λ = 0.71073 Å
*b* = 9.4790 (2) Å	Cell parameters from 6293 reflections
*c* = 10.4939 (2) Å	θ = 2.2–27.5°
α = 67.396 (1)°	µ = 2.60 mm^−^^1^
β = 89.933 (1)°	*T* = 173 K
γ = 82.373 (1)°	Block, colourless
*V* = 838.74 (3) Å^3^	0.39 × 0.33 × 0.22 mm

### Data collection

**Table:**

Bruker SMART APEXII CCD diffractometer	3888 independent reflections
Radiation source: rotating anode	3417 reflections with *I* > 2σ(*I*)
Graphite multilayer monochromator	*R*_int_ = 0.045
Detector resolution: 10.0 pixels mm^-1^	θ_max_ = 27.6°, θ_min_ = 2.1°
φ and ω scans	*h* = −12→12
Absorption correction: multi-scan (*SADABS*; Bruker, 2009)	*k* = −12→12
*T*_min_ = 0.557, *T*_max_ = 0.746	*l* = −13→13
15136 measured reflections	

### Refinement

**Table:**

Refinement on *F*^2^	Primary atom site location: structure-invariant direct methods
Least-squares matrix: full	Secondary atom site location: difference Fourier map
*R*[*F*^2^ > 2σ(*F*^2^)] = 0.030	Hydrogen site location: difference Fourier map
*wR*(*F*^2^) = 0.079	H-atom parameters constrained
*S* = 1.05	*w* = 1/[σ^2^(*F*_o_^2^) + (0.032*P*)^2^ + 0.4488*P*] where *P* = (*F*_o_^2^ + 2*F*_c_^2^)/3
3888 reflections	(Δ/σ)_max_ = 0.001
226 parameters	Δρ_max_ = 0.65 e Å^−^^3^
0 restraints	Δρ_min_ = −0.74 e Å^−^^3^

### Special details

**Table:**

Geometry. All esds (except the esd in the dihedral angle between two l.s. planes) are estimated using the full covariance matrix. The cell esds are taken into account individually in the estimation of esds in distances, angles and torsion angles; correlations between esds in cell parameters are only used when they are defined by crystal symmetry. An approximate (isotropic) treatment of cell esds is used for estimating esds involving l.s. planes.
Refinement. Refinement of F^2^ against ALL reflections. The weighted R-factor wR and goodness of fit S are based on F^2^, conventional R-factors R are based on F, with F set to zero for negative F^2^. The threshold expression of F^2^ > 2sigma(F^2^) is used only for calculating R-factors(gt) etc. and is not relevant to the choice of reflections for refinement. R-factors based on F^2^ are statistically about twice as large as those based on F, and R- factors based on ALL data will be even larger.

### Fractional atomic coordinates and isotropic or equivalent isotropic displacement parameters (Å^2^)

**Table:**

	*x*	*y*	*z*	*U*_iso_*/*U*_eq_	
Br1	0.44656 (2)	1.17299 (2)	0.60200 (2)	0.03743 (9)	
S1	0.34716 (5)	0.48730 (6)	0.74923 (5)	0.02461 (11)	
F1	−0.06628 (17)	0.76294 (19)	0.26600 (15)	0.0505 (4)	
O1	0.25847 (16)	0.62748 (16)	1.05071 (14)	0.0266 (3)	
O2	0.49317 (16)	0.5237 (2)	0.69525 (17)	0.0363 (4)	
C1	0.2987 (2)	0.5932 (2)	0.8526 (2)	0.0234 (4)	
C2	0.3270 (2)	0.7466 (2)	0.8310 (2)	0.0241 (4)	
C3	0.3706 (2)	0.8705 (2)	0.7217 (2)	0.0267 (4)	
H3	0.3888	0.8656	0.6343	0.032*	
C4	0.3862 (2)	1.0009 (2)	0.7466 (2)	0.0282 (4)	
C5	0.3610 (2)	1.0122 (3)	0.8731 (2)	0.0321 (5)	
H5	0.3731	1.1046	0.8848	0.039*	
C6	0.3183 (2)	0.8897 (3)	0.9821 (2)	0.0314 (5)	
H6	0.3012	0.8946	1.0697	0.038*	
C7	0.3020 (2)	0.7600 (2)	0.9568 (2)	0.0255 (4)	
C8	0.2584 (2)	0.5276 (2)	0.9848 (2)	0.0240 (4)	
C9	0.2165 (2)	0.3774 (2)	1.0659 (2)	0.0247 (4)	
C10	0.2675 (2)	0.2991 (2)	1.2042 (2)	0.0288 (4)	
H10	0.3274	0.3452	1.2461	0.035*	
C11	0.2304 (3)	0.1551 (3)	1.2792 (2)	0.0367 (5)	
H11	0.2672	0.1007	1.3723	0.044*	
C12	0.1402 (3)	0.0891 (3)	1.2200 (3)	0.0421 (6)	
H12	0.1140	−0.0097	1.2729	0.051*	
C13	0.0878 (3)	0.1664 (3)	1.0836 (3)	0.0403 (5)	
H13	0.0256	0.1209	1.0432	0.048*	
C14	0.1259 (2)	0.3097 (3)	1.0066 (2)	0.0330 (5)	
H14	0.0905	0.3624	0.9129	0.040*	
C15	0.2184 (2)	0.5861 (2)	0.60419 (19)	0.0227 (4)	
C16	0.0772 (2)	0.6487 (3)	0.6157 (2)	0.0299 (4)	
H16	0.0468	0.6502	0.7017	0.036*	
C17	−0.0195 (2)	0.7090 (3)	0.5006 (2)	0.0351 (5)	
H17	−0.1168	0.7531	0.5060	0.042*	
C18	0.0288 (2)	0.7034 (3)	0.3788 (2)	0.0328 (5)	
C19	0.1680 (3)	0.6428 (3)	0.3644 (2)	0.0320 (5)	
H19	0.1977	0.6420	0.2779	0.038*	
C20	0.2640 (2)	0.5827 (2)	0.4797 (2)	0.0267 (4)	
H20	0.3612	0.5391	0.4735	0.032*	

### Atomic displacement parameters (Å^2^)

**Table:**

	*U*^11^	*U*^22^	*U*^33^	*U*^12^	*U*^13^	*U*^23^
Br1	0.04254 (14)	0.02593 (13)	0.04180 (14)	−0.00937 (9)	0.01229 (10)	−0.00950 (10)
S1	0.0270 (2)	0.0261 (3)	0.0238 (2)	−0.00184 (18)	0.00172 (18)	−0.0136 (2)
F1	0.0572 (9)	0.0564 (10)	0.0337 (7)	0.0041 (7)	−0.0179 (7)	−0.0169 (7)
O1	0.0355 (8)	0.0270 (7)	0.0204 (7)	−0.0083 (6)	0.0049 (5)	−0.0113 (6)
O2	0.0255 (7)	0.0541 (10)	0.0394 (9)	−0.0050 (7)	0.0052 (6)	−0.0293 (8)
C1	0.0251 (9)	0.0254 (10)	0.0215 (9)	−0.0040 (7)	0.0017 (7)	−0.0112 (8)
C2	0.0239 (9)	0.0264 (10)	0.0241 (9)	−0.0031 (7)	0.0015 (7)	−0.0123 (8)
C3	0.0288 (10)	0.0271 (10)	0.0246 (10)	−0.0045 (8)	0.0052 (8)	−0.0104 (8)
C4	0.0258 (10)	0.0253 (10)	0.0318 (10)	−0.0049 (8)	0.0037 (8)	−0.0090 (9)
C5	0.0362 (11)	0.0275 (11)	0.0369 (12)	−0.0054 (9)	0.0012 (9)	−0.0170 (10)
C6	0.0402 (12)	0.0327 (12)	0.0274 (10)	−0.0070 (9)	0.0035 (9)	−0.0176 (9)
C7	0.0279 (10)	0.0267 (10)	0.0231 (9)	−0.0060 (8)	0.0034 (7)	−0.0103 (8)
C8	0.0243 (9)	0.0276 (10)	0.0231 (9)	−0.0043 (7)	−0.0002 (7)	−0.0130 (8)
C9	0.0247 (9)	0.0258 (10)	0.0250 (9)	−0.0042 (7)	0.0047 (7)	−0.0112 (8)
C10	0.0281 (10)	0.0293 (11)	0.0279 (10)	−0.0018 (8)	0.0026 (8)	−0.0107 (9)
C11	0.0433 (12)	0.0293 (12)	0.0298 (11)	−0.0002 (9)	0.0084 (9)	−0.0048 (9)
C12	0.0493 (14)	0.0257 (12)	0.0510 (15)	−0.0111 (10)	0.0186 (11)	−0.0126 (11)
C13	0.0401 (13)	0.0371 (13)	0.0515 (15)	−0.0134 (10)	0.0071 (11)	−0.0233 (12)
C14	0.0324 (11)	0.0363 (12)	0.0341 (11)	−0.0072 (9)	0.0014 (9)	−0.0170 (10)
C15	0.0267 (9)	0.0223 (9)	0.0208 (9)	−0.0064 (7)	0.0022 (7)	−0.0093 (8)
C16	0.0285 (10)	0.0374 (12)	0.0265 (10)	−0.0041 (8)	0.0047 (8)	−0.0155 (9)
C17	0.0296 (11)	0.0419 (13)	0.0331 (11)	−0.0004 (9)	−0.0009 (9)	−0.0154 (10)
C18	0.0405 (12)	0.0299 (11)	0.0263 (10)	−0.0058 (9)	−0.0070 (9)	−0.0087 (9)
C19	0.0452 (12)	0.0314 (11)	0.0215 (10)	−0.0089 (9)	0.0060 (9)	−0.0116 (9)
C20	0.0298 (10)	0.0261 (10)	0.0262 (10)	−0.0059 (8)	0.0072 (8)	−0.0118 (9)

### Geometric parameters (Å, º)

**Table:**

Br1—C4	1.900 (2)	C9—C10	1.400 (3)
S1—O2	1.4923 (16)	C10—C11	1.378 (3)
S1—C1	1.7609 (19)	C10—H10	0.9500
S1—C15	1.7941 (19)	C11—C12	1.382 (4)
F1—C18	1.359 (2)	C11—H11	0.9500
O1—C8	1.371 (2)	C12—C13	1.385 (4)
O1—C7	1.375 (2)	C12—H12	0.9500
C1—C8	1.358 (3)	C13—C14	1.380 (3)
C1—C2	1.443 (3)	C13—H13	0.9500
C2—C7	1.390 (3)	C14—H14	0.9500
C2—C3	1.394 (3)	C15—C20	1.383 (3)
C3—C4	1.382 (3)	C15—C16	1.383 (3)
C3—H3	0.9500	C16—C17	1.385 (3)
C4—C5	1.389 (3)	C16—H16	0.9500
C5—C6	1.382 (3)	C17—C18	1.371 (3)
C5—H5	0.9500	C17—H17	0.9500
C6—C7	1.380 (3)	C18—C19	1.369 (3)
C6—H6	0.9500	C19—C20	1.383 (3)
C8—C9	1.454 (3)	C19—H19	0.9500
C9—C14	1.396 (3)	C20—H20	0.9500
			
O2—S1—C1	107.36 (9)	C11—C10—H10	120.1
O2—S1—C15	105.38 (9)	C9—C10—H10	120.1
C1—S1—C15	100.79 (9)	C10—C11—C12	120.5 (2)
C8—O1—C7	106.44 (15)	C10—C11—H11	119.8
C8—C1—C2	107.19 (17)	C12—C11—H11	119.8
C8—C1—S1	123.20 (15)	C11—C12—C13	120.2 (2)
C2—C1—S1	127.98 (15)	C11—C12—H12	119.9
C7—C2—C3	119.21 (18)	C13—C12—H12	119.9
C7—C2—C1	104.74 (18)	C14—C13—C12	120.0 (2)
C3—C2—C1	136.05 (18)	C14—C13—H13	120.0
C4—C3—C2	116.90 (19)	C12—C13—H13	120.0
C4—C3—H3	121.6	C13—C14—C9	120.2 (2)
C2—C3—H3	121.6	C13—C14—H14	119.9
C3—C4—C5	123.2 (2)	C9—C14—H14	119.9
C3—C4—Br1	118.81 (16)	C20—C15—C16	120.94 (18)
C5—C4—Br1	118.02 (16)	C20—C15—S1	115.41 (15)
C6—C5—C4	120.30 (19)	C16—C15—S1	123.34 (15)
C6—C5—H5	119.8	C15—C16—C17	119.43 (19)
C4—C5—H5	119.8	C15—C16—H16	120.3
C7—C6—C5	116.44 (19)	C17—C16—H16	120.3
C7—C6—H6	121.8	C18—C17—C16	118.3 (2)
C5—C6—H6	121.8	C18—C17—H17	120.9
O1—C7—C6	125.29 (18)	C16—C17—H17	120.9
O1—C7—C2	110.73 (17)	F1—C18—C19	118.31 (19)
C6—C7—C2	124.0 (2)	F1—C18—C17	118.2 (2)
C1—C8—O1	110.88 (17)	C19—C18—C17	123.52 (19)
C1—C8—C9	133.00 (18)	C18—C19—C20	117.88 (19)
O1—C8—C9	116.11 (17)	C18—C19—H19	121.1
C14—C9—C10	119.4 (2)	C20—C19—H19	121.1
C14—C9—C8	120.44 (19)	C15—C20—C19	119.95 (19)
C10—C9—C8	120.11 (18)	C15—C20—H20	120.0
C11—C10—C9	119.7 (2)	C19—C20—H20	120.0
			
O2—S1—C1—C8	−127.11 (17)	C7—O1—C8—C9	179.50 (16)
C15—S1—C1—C8	122.87 (17)	C1—C8—C9—C14	−41.0 (3)
O2—S1—C1—C2	36.4 (2)	O1—C8—C9—C14	138.82 (19)
C15—S1—C1—C2	−73.58 (19)	C1—C8—C9—C10	138.7 (2)
C8—C1—C2—C7	0.7 (2)	O1—C8—C9—C10	−41.5 (3)
S1—C1—C2—C7	−164.98 (15)	C14—C9—C10—C11	1.4 (3)
C8—C1—C2—C3	−179.8 (2)	C8—C9—C10—C11	−178.28 (19)
S1—C1—C2—C3	14.5 (3)	C9—C10—C11—C12	−1.7 (3)
C7—C2—C3—C4	−0.1 (3)	C10—C11—C12—C13	0.9 (4)
C1—C2—C3—C4	−179.5 (2)	C11—C12—C13—C14	0.1 (4)
C2—C3—C4—C5	0.2 (3)	C12—C13—C14—C9	−0.4 (3)
C2—C3—C4—Br1	179.60 (14)	C10—C9—C14—C13	−0.4 (3)
C3—C4—C5—C6	0.1 (3)	C8—C9—C14—C13	179.3 (2)
Br1—C4—C5—C6	−179.34 (16)	O2—S1—C15—C20	40.06 (17)
C4—C5—C6—C7	−0.5 (3)	C1—S1—C15—C20	151.62 (15)
C8—O1—C7—C6	−179.2 (2)	O2—S1—C15—C16	−146.37 (18)
C8—O1—C7—C2	1.1 (2)	C1—S1—C15—C16	−34.81 (19)
C5—C6—C7—O1	−179.01 (19)	C20—C15—C16—C17	−0.2 (3)
C5—C6—C7—C2	0.6 (3)	S1—C15—C16—C17	−173.42 (17)
C3—C2—C7—O1	179.31 (17)	C15—C16—C17—C18	0.4 (3)
C1—C2—C7—O1	−1.1 (2)	C16—C17—C18—F1	−179.9 (2)
C3—C2—C7—C6	−0.4 (3)	C16—C17—C18—C19	−0.6 (4)
C1—C2—C7—C6	179.24 (19)	F1—C18—C19—C20	179.91 (19)
C2—C1—C8—O1	0.0 (2)	C17—C18—C19—C20	0.7 (4)
S1—C1—C8—O1	166.47 (13)	C16—C15—C20—C19	0.2 (3)
C2—C1—C8—C9	179.8 (2)	S1—C15—C20—C19	173.95 (16)
S1—C1—C8—C9	−13.7 (3)	C18—C19—C20—C15	−0.4 (3)
C7—O1—C8—C1	−0.6 (2)		

### Hydrogen-bond geometry (Å, º)

**Table:**

*D*—H···*A*	*D*—H	H···*A*	*D*···*A*	*D*—H···*A*
C10—H10···O2^i^	0.95	2.41	3.341 (3)	167
C19—H19···O1^ii^	0.95	2.50	3.447 (2)	175
C16—H16···*Cg*^iii^	0.95	2.99	3.832 (2)	148

Symmetry codes: (i) −*x*+1, −*y*+1, −*z*+2; (ii) *x*, *y*, *z*−1; (iii) −*x*, −*y*+1, −*z*+2.
